# Synergic Efficacy of a Multicomponent Nutraceutical Add-On Therapy in Seasonal Allergic Rhinitis in Children: A Prospective, Randomized, Parallel-Group Study

**DOI:** 10.3390/jcm14051517

**Published:** 2025-02-24

**Authors:** Alessandra Gori, Giulia Brindisi, Caterina Anania, Alberto Spalice, Anna Maria Zicari

**Affiliations:** Department of Mother-Child, Urological Science, La Sapienza University, 00161 Rome, Italy; giulia.brindisi@uniroma1.it (G.B.); caterina.anania@uniroma1.it (C.A.); alberto.spalice@uniroma1.it (A.S.); annamaria.zicari@uniroma1.it (A.M.Z.)

**Keywords:** allergic rhinitis (AR), nutraceuticals, Quercetin, *Perilla frutescens*, vitamin D3, nasal nitric oxide, exhaled nitric oxide, nasal cytology, randomized controlled trial (RCT), pediatric allergy

## Abstract

**Background:** Emerging evidence suggests that nutraceuticals, alongside standard therapy, may benefit children with allergic rhinitis (AR). This study aimed to compare the efficacy of Quertal^®^ (Neopharmed Gentili S.p.A., Milano, Italy), a nutraceutical supplement based on *Perilla frutescens*, Quercetin, and vitamin D3, combined antihistamines per os versus antihistamines alone, in improving AR symptoms considering respiratory functional and laboratory biomarkers in pediatric age. **Materials and Method:** This study included 100 children, 50 in the case group (Quertal^®^ plus antihistamines) and 50 in the control group (antihistamines alone), with mild/moderate AR sensitized to grass pollens. They underwent assessments of respiratory function (rhinomanometry-AAR, spirometry), inflammation markers (Nasal Nitric Oxide [nFeNO]; exhaled Nitric Oxide [eFeNO]; nasal cytology), and laboratory assays (blood eosinophils, total IgE and specific IgE to Phl p1). **Results:** After three months of treatment, the case group showed statistically significant improvement in nFeNO and eFeNO values compared to controls (*p* < 0.001), as well as a reduction in nasal eosinophils (*p* < 0.001). **Conclusions:** Adding Quertal^®^ to standard antihistamine therapy may reduce nasal inflammation and improve AR symptoms in pediatric patients. This combination therapy shows promise as a practical, well-tolerated approach to managing AR and may have broader implications for enhancing long-term outcomes.

## 1. Introduction

Allergic rhinitis (AR) is a prevalent chronic respiratory disease affecting up to 50% of the global population, particularly in industrialized countries. It poses a significant public health concern due to its social and economic impact [1]. In children, AR manifests with high frequency (from 10% to 30% incidence [2], while prevalence rises as children grow older, from 8.5% to 14.6% (ISAAC) [3,4]).

The delayed diagnosis or improper management can lead to the persistence and progression of symptoms from the upper to the lower respiratory tract, ultimately causing chronic conditions [3,5,6].

Despite advancements in understanding AR’s pathogenesis, based on classic Type 2 inflammation that involved a complex architecture of cellular and humoral mediators enabling the cascading recruitment and bridging of innate and adaptive immunity [7,8,9], there remains a pressing need for innovation in treatment options. Current management largely depends on symptomatic therapy, such as antihistamines and corticosteroids, and Allergen-specific Immunotherapy (AIT), the only disease-modifying management option [10]. International researchers’ interest in the bioactive principles of complementary medicine has led to the utilization of nutraceuticals, offering new therapeutic possibilities. This approach is based on applying current knowledge on the pathogenesis of diseases and verifying the efficacy of such compounds through scientific methods and a careful process of validation, transformation, and production [11].

Among the most notable nutraceuticals mentioned in the literature in this field, Quertal^®^ leverages bioactive compounds from food and medicinal plants to target the inflammatory pathways of AR, potentially enhancing the efficacy of standard treatments while reducing their side effects. Containing *Perilla frutescens*, Quercetin, and vitamin D3, this supplement stands out for its anti-inflammatory, antioxidant, and immunomodulatory properties due to its unique composition [12,13,14]. In particular, in *Perilla frutescens*, 271 active compounds have been identified in the seeds, stems, and leaves, including rosmarinic acid and luteolin with anti-allergic (antihistaminic with inhibition of mast cell degranulation); antioxidant (scavenging of ROSs (suppression of inducible nitric oxide synthase (iNOS) expression)); and anti-inflammatory (reduction in the release of the HMGB1 protein with an inhibitory effect on pro-inflammatory cytokines (TNF-α and IL-6) and Nitric Oxide) properties [12,15,16].

Quercetin is the most abundant dietary flavonoid, present in a wide range of fruits, vegetables, and beverages, with high interest and success in the literature (currently over 23,000 citations in PubMed). From this vast number of data, quercetin improves pathological conditions involving inflammation and oxidative stress at different levels of the allergic inflammatory cascade by acting on cellular and humoral targets throughout the respiratory tree. In particular, Quercetin reduces airway allergic inflammation and hyperreactivity caused by the Th1/Th2 imbalance and has a suppressive effect on the cells’ ability to produce CCL5, eotaxin, periostin, and NO, leading to an improvement in the clinical condition of patients with AR [14,17,18,19,20,21].

Indeed, the last compound is vitamin D3. Its role suggests a dynamic process that begins in the womb and continues throughout childhood and probably into adulthood, during which vitamin D plays a critical role in interfering with the immunopathogenic mechanisms of respiratory allergy development. The increase in allergic diseases has coincided with the frequent deficiency represented in many populations of vitamin D, and this inverse relationship has raised the idea of a possible underlying association between these two opposite trends [13].

Vitamin D impacts both innate and adaptive immunity by inhibiting Toll-like receptors on monocytes, reducing proinflammatory cytokines, and promoting antimicrobial peptides. It influences T and B lymphocytes, reducing IgE secretion, balancing Th1/Th2 lymphocytes, and supporting regulatory T cells (Treg). Vitamin D deficiency can shift this balance, leading to increased Th2 response. It also affects airway remodeling and maintains nasal epithelium integrity, protecting against allergens. Studies show that vitamin D enhances the response to dexamethasone by improving IL-10 production and reducing steroid resistance in asthma [22].

Thus, this study aims to evaluate the potential of non-pharmacological treatments to complement conventional therapies in managing AR in children. By using tools like anterior active rhinomanometry (AAR), spirometry, nasal and exhaled nitric oxide (nFeNO, eFeNO), nasal cytology, and laboratory tests (eosinophils, total and specific IgE levels), it provides evidence for improved prevention, particularly in secondary and tertiary care, offering tailored therapeutic strategies for pediatric patients.

## 2. Materials and Methods

### 2.1. Study Design

This study followed a prospective, randomized, parallel-group design, comparing two groups of children with mild/moderate AR sensitized exclusively to grass pollen (*Phleum pretense*). The case group received Quertal^®^ in addition to oral antihistamines, while the control group received only antihistamines. The study was conducted at the Pediatric Allergology Service of Policlinico Umberto I, the Sapienza University of Rome, from February to April 2023, lasting approximately 12 weeks for each patient. This study was conducted following the CONSORT (Consolidated Standards of Reporting Trials) guidelines to ensure high methodological quality and transparent reporting. The corresponding CONSORT checklist has been completed and is available in Supplementary Table S1.

### 2.2. Study Population

The inclusion criteria included age from 6 to 16 years, diagnosis of AR with sensitization to grass pollen (*Phleum pretense*) only with positive SPTs (wheals ≥ 3 mm) and sIgE to Phl p1, the major grass pollen allergen [23] (>0.35 KU/L), residence in the metropolitan area of Rome, written informed consent from the patient and the parent or legal guardian.

The exclusion criteria were coexistence of chronic diseases (such as uncontrolled asthma, neurological disorders, genetic syndromes, neuromuscular disorders, or medications that depress the respiratory system), inadequate washout of medications (systemic or intranasal corticosteroids: 1 month; Leukotriene antagonists: 1 month; systemic or intranasal decongestants: 3 days), upper or lower respiratory infection in the last two weeks, participation in other clinical studies in the previous month, and present or past allergen immunotherapy (AIT).

In total, 125 children were initially enrolled, but 25 patients dropped out, leaving 100 participants: 50 in the case group and 50 in the control group.

### 2.3. Randomization

Participants were randomly assigned to either the case group (receiving Quertal^®^ plus antihistamines) or the control group (receiving antihistamines only) using a computer-generated randomization sequence. To ensure balance across age, sex, and ethnicity, participants were divided into strata, and randomization was performed within each subgroup.

Each participant was assigned a unique identification number upon enrollment, and the randomization followed a 1:1 ratio, ensuring that each participant had an equal chance of being allocated to either group. Random numbers were generated using SPSS software 27.0 (IBM, Chicago, IL, USA) and the list of participants was sorted based on these numbers. Participants were then sequentially assigned to the case or control group. To maintain allocation concealment and prevent selection bias, the randomization sequence was sealed in opaque, sequentially numbered envelopes. These envelopes were opened only after the participant’s enrollment was complete.

### 2.4. Study Phases

1. T0-Enrollment phase: This phase involved allergological visit and demographic and anamnestic data following inclusion/exclusion criteria. Then, a physical examination, a clinical evaluation with the classification of AR according to ARIA criteria, and Skin Prick Tests (SPTs) were performed. Suitable patients were then allocated in a 1:1 ratio into two groups: The case group treated with Quertal^®^ double-layer tablets (1 tablet per day for three months) plus oral antihistamines therapy, and the control group treated with oral antihistamines only.

2. T1-Start of therapy: One week approximately after T0, a clinical evaluation was conducted at a day hospital (DH), where laboratory and instrumental tests were performed. Therapy was prescribed (Quertal^®^ plus antihistamines for the case group, antihistamines only for the control group), and patients were given clinical diaries to record symptoms, occurrence/duration of exacerbations, and adverse events.

3. T2-Follow-up: After three months of therapy administration, another clinical evaluation was conducted at the DH, repeating the laboratory and instrumental tests performed at T1.

### 2.5. Study Outcomes

The study outcomes were classified into primary and secondary measures to ensure a comprehensive evaluation of treatment efficacy. The primary outcomes included changes in nFeNO, eFeNO, nasal cytology, and NSS scores, assessed at T0 (baseline) and T2 (12 weeks). The secondary outcomes encompassed alterations in serological biomarkers, including the eosinophil count, total IgE, and specific IgE to Phl p1, measured through standardized immunoassay techniques. These endpoints were chosen to provide both subjective symptom assessment (NSS) and objective biomarkers of inflammation and airway function.

### 2.6. Serological Biomarkers

Serum total IgE levels and specific molecular IgE levels to Phleum pratense (Phl P1) were tested using a fluorescence enzyme immunoassay (FEIA) with capsulated cellulose polymer solid phase (Immuno CAP^®^) coupled allergens (Thermo Fisher Scientific Inc., Phadia AB, Uppsala, Sweden). Results were expressed in kU/L, and a cut-off point of 0.35 kU/L stands for positivity. We also tested the blood eosinophil count.

### 2.7. Nasal Eosinophils Count and C/G Ratio at Nasal Cytology

Nasal cytology was performed by a small plastic curette (nasal scraping) at the middle portion of the inferior turbinate. Then, the cellular material was spread on a glass slide, fixed by air drying, and stained following the May–Grunwald Giemsa (MGG) guideline. After these procedures, it was possible to evaluate nasal mucosa composition with the cells of immune-phlogosis (neutrophils, eosinophils, lymphocytes, and mast cells), bacteria, mycotic spores, and fungal hyphae thanks to a standard optical microscope at 1000X. In the rhino-cytogram, mainly, we searched nasal eosinophils reading by fields (minimum 50). The ratio of ciliated cells to goblet cells (C/G ratio) was assessed by counting the number of each cell type in five high-power fields (HPFs) under light microscopy (magnification × 400). Goblet cells were identified by their mucin-filled cytoplasm, while ciliated cells were distinguished by their apical cilia. The average ratio of goblet cells to ciliated cells was calculated for each sample to evaluate mucociliary alteration associated with allergic rhinitis [24]. We excluded patients receiving local nasal steroid therapy precisely to assess changes in nasal cytology (dropout).

### 2.8. Nasal Nitric Oxide (nFeNO)

Nasal FeNO (nFeNO) was performed in spontaneous breathing and using a tight facemask with a fixed flow connected to an analyzer (Medisoft nFeNO analyzer, Medisoft s.r.l., Naples, Italy). The measurement of nFeNO was made according to the American Thoracic Society/European Respiratory Society guidelines’ values (ERS/ATS). During inspiration up to total lung capacity, patients inhaled through the nose from the NO-free analyzer and exhaled through disposable nose pads at a constant flow of 350 mL/s for 60 s [25,26].

### 2.9. Exhaled Nitric Oxide (eFeNO)

Exhaled Nitric Oxide (eFeNO) was measured with a Cosmed Quark NO breath device following the ATS/ERS procedures published for eFeNO measurement [27].

### 2.10. Mean Nasal Flow (mNF) at Active Anterior Rhinomanometry (AAR)

AAR detected mean nasal flow (mNF) and nasal resistance to the air passage, taking about 10 min. It was performed according to the ICSR (Committee for the Standardization of Rhinomanometry) guidelines’ values using a RINOPOCKET ED200 (EUROCLINIC^®^, Imola, Bologna, Italy). Thanks to this objective method, we measured the nasal flow (cm^3^/s) in the right and left nostrils at a pressure of 150 Pa in both inspiration and expiration [28]. The obtained values were then compared with the pediatric ones’, adjusted for height. Nasal obstruction is classified as very severe if airflow values are less than 29% of the predicted ones, severe if the values are between 29% and 42%, moderate if between 43% and 56%, mild between 57% and 70%, and absent for values above 71% [29]. Patients also performed an AAR post-decongestion to provide additional information using a sympathetic alpha mimetic such as Naphazoline Nitrate nasal spray (Rinazine^®^ (Haleon Italy S.r.l., Milano, Italy) 100 mg/100 mL), one puff in each nostril. After 15 min, an AAR was repeated, evaluating the possible modification of the nasal airflow and resistance.

### 2.11. Forced Expiratory Volume in the First Second (FEV1) at Spirometry

FEV1 was used to detect bronchial obstruction through spirometry, measured with a Cosmed Spirometer (Cosmed, Rome, Italy), according to ATS/ERS procedures. Basal FEV1 was expressed as a percentage of predicted average values adjusted for height, sex, and ethnicity. Patients also performed a spirometry post-bronchodilatation with Salbutamol spray 100 mcg, according to ATS/ERS procedures [30].

### 2.12. Nasal Symptom Score (NSS)

NSS is a patient-reported evaluation made by four items to assess symptoms, including nasal obstruction, rhinorrhea, itching, and sneezing. The answer to each item presents a score ranging from 0 to 3 (0, absent; 1, weak; 2, moderate; 3, severe). The sum of the total indicates the severity of rhinitis with a maximum score of 12. At baseline (T1) and (T2), patients were asked to assign a score to each symptom experienced in the last 12 h and over the previous two weeks.

### 2.13. Treatment Compliance, Rescue Medication, and Adverse Events

Patients were instructed to return all unused Quertal^®^ and antihistamine tablets at the T2 visit. Compliance was assessed by counting the remaining tablets subsequently returned to the patient. Both patients and their parents were advised to inform the investigators of any exacerbation symptoms and rescue or concomitant therapy. Mainly, the use of systemic or intranasal corticosteroids, leukotriene antagonists, and other intranasal medications resulted in exclusion from the study. Any adverse event—defined as any unexpected medical occurrence in a patient receiving Quertal^®^, which does not necessarily have a causal relationship with the treatment—was documented, and appropriate measures for patient safety and follow-up were taken.

### 2.14. Schedule of Quertal^®^ Administration

Regarding the method of administration, each patient of the “case group” took one tablet of Quertal^®^ a day, administered together with antihistamine therapy for three months.

Each tablet had a dual layer for fast Perilla and slow Quercetin/vitamin D3 release. The specific content of the active ingredients is 80 mg for *Perilla frutescens*, 150 mg for Quercetin, and 5 mcg (200 IU) for vitamin D3.

### 2.15. Standard Treatment

Standard therapy was an antihistamine chosen by the investigator, considering the specific needs of the patients. Generally, Cetirizine or Levocetirizine were the preferable antihistamine molecules.

## 3. Statistical Analysis

The sample size was determined based on a power calculation to detect a clinically significant difference in nasal nitric oxide levels (nFeNO) between groups. A hundred patients (50 per group) were required to achieve an 80% power with an alpha level of 0.05. This calculation was based on previous studies assessing similar nutraceutical interventions in allergic rhinitis. Statistical analysis was performed using IBM SPSS version 27.0 (SPSS, Chicago, IL, USA). For the continuous variables, the Shapiro–Wilk normality test was performed; these continuous quantities were represented by the mean value and standard deviation (SD). Nominal and ordinal variables were described in terms of relative counts and frequencies. The comparison of the assumed clinical values—both for the case and control groups—was performed by “paired-samples T-test” and by “Wilcoxon signed-rank test for paired samples”. The comparison between the case and control groups, both at T0 and at T1, was performed by the “*t*-test for unpaired samples” and the “U Mann–Whitney test”. In all cases, a *p*-value ≤ 0.05 was considered statistically significant.

## 4. Results

The characteristics of the study population are reported in Table 1 below. No significant differences were noted regarding age, weight, height, and BMI between the case and control groups. The associated presence of mild intermittent asthma between the case and control groups is reported in Table 1.

None of the patients with mild intermittent asthma underwent emergency therapy (salbutamol and inhaled corticosteroid) due to the presence of an asthma attack during the study.

All patients demonstrated compliance with the therapy, both those in the case group and those in the control group, except seven patients who missed one or two doses during the entire treatment period, three patients in the case group, and four in the control group. No patient experienced adverse effects or clinical manifestations attributable to the administration of the supplement.

After the descriptive analysis of the data, we conducted an intra- and inter-group inferential analysis (Table 2).

**Figure 1:**
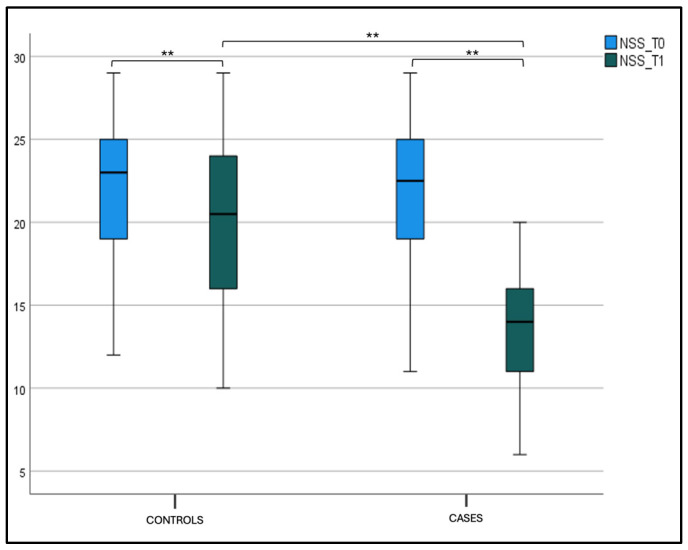
Nasal symptom score (NSS) at T0 and T1 in the case and control groups. Box plot representing NSS values at T0 and T1 in both the case and control groups (** significant).

Considering the NSS intragroup variability both for the case and control groups, we found a statistically significant difference between T0 and T1 values (*p* < 0.001). The same significance for the NSS intergroup variability at T1 (*p* < 0.001) was found, as shown in the box plot Figure 1 and Table 2.

**Figure 2:**
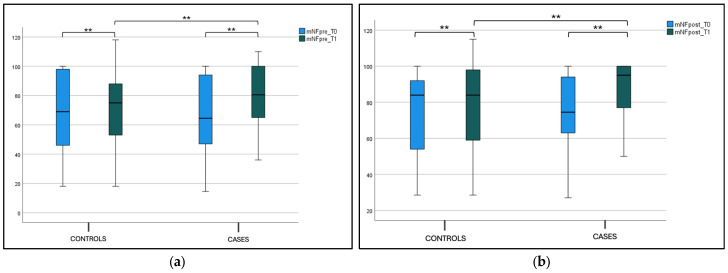
(**a**) Mean nasal flow (mNF) pre-hydrazine at T0 and T1 in the case and control groups. Box plot representing mean nasal flow (mNF) pre-hydrazine at T0 and T1 in both case and control groups (** significant). (**b**) Mean nasal flow (mNF) post-hydrazine at T0 and T1 in the case and control groups. Box plot representing mean nasal flow (mNF) post-hydrazine at T0 and T1 in both case and control groups (** significant).

Considering mNF pre-hydrazine and mNF post-hydrazine, we found statistical significance for the intragroup variability both for the case and control groups, between T0 and T1 values (*p* < 0.001). The same significance was found for the intergroup variability at T1, as shown in Figure 2a,b and Table 2.

**Figure 3:**
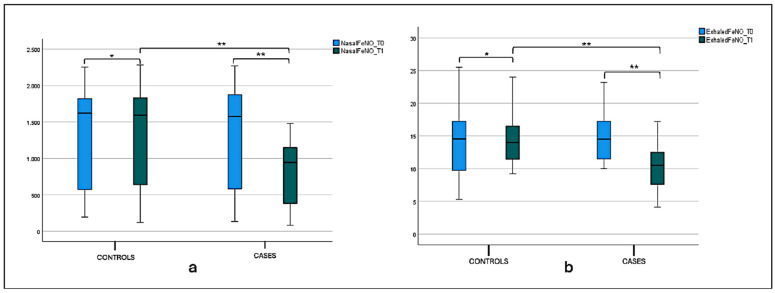
(**a**) Nasal FeNO (nFeNO) at T0 and T1 in the treatment and control groups. Box plot representing nFeNO values at T0 and T1 in both the treatment and control groups (* not significant, ** significant). (**b**) Exhaled FeNO (eFeNO) at T0 and T1 in the treatment and control groups. Box plot representing eFeNO values at T0 and T1 in both the treatment and control groups (* not significant, ** significant).

A statistical significance difference between nFeN0 at T0 (1277.5 ± 697.21) and T1 (798.1 ± 436.21) (*p* < 0.001) was observed in the case group, as shown in Figure 3, and Table 2 Considering the intergroup variability, we did not find a statistically significant difference between the nFeNO of the case and control groups at T0; this difference was found at T1 (*p* < 0.001), as shown in Figure 3a and Table 2. The eFeNO values in the case group showed a statistical significance difference between eFeN0 at T0 (14.86 ± 3.79) and T1 (10.33 ± 3.36) (*p* < 0.001), as shown in Figure 3b and Table 1. Considering the intergroup variability, we did not find a statistically significant difference between the eFeNO of the case and control groups at T0; this difference was found at T1 (*p* < 0.001), as shown in Figure 3b and Table 2.

**Figure 4:**
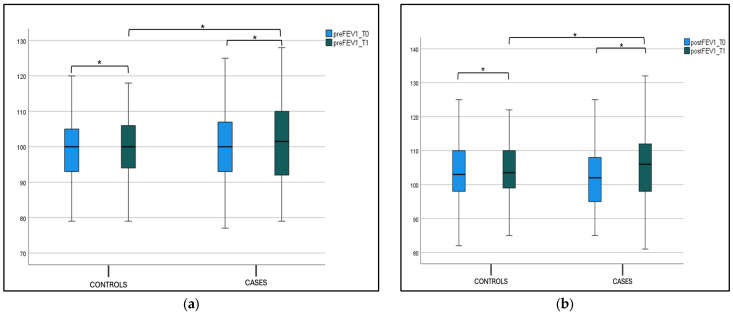
(**a**) FEV1 pre-bronchodilatation (preFEV1) at T0 and T1 in the case and control groups. Box plot representing preFEV1 values at T0 and T1 in both the case and control groups (* not significant). (**b**) FEV1 post-bronchodilatation (postFEV1) at T0 and T1 in the case and control groups. Box plot representing postFEV1 values at T0 and T1 in both the case and control groups (* not significant).

Instead, we did not find a statistically significant difference in both preFEV1 and postFEV1 values between the case and control groups at T1 (p-value intergroup). This difference was not found, respectively, for “preFEV1” and “postFEV1” intragroup values, as shown in Figure 4a,b and Table 2.

**Figure 5:**
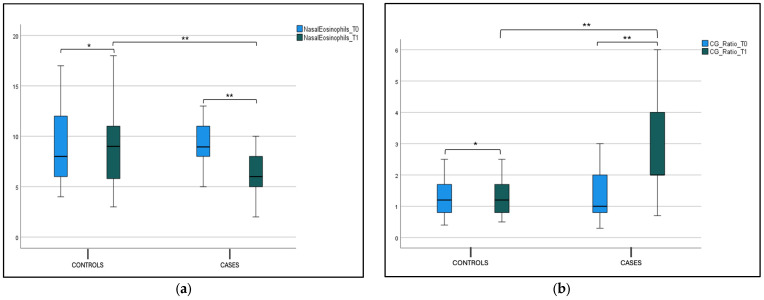
(**a**) Nasal eosinophils at T0 and T1 in the case and control group. Box plot representing nasal eosinophils at cytology at T0 and T1 in both case and control group (* not significant, ** significant). (**b**) C/G ratio at T0 and T1 in the case and control group. Box plot representing C/G ratio at T0 and T1 in both case and control group (* not significant, ** significant).

Considering the percentage of eosinophil cells in nasal cytology, we found a statistical significance at T0 (9.20 ± 2.16) and T1 (6.20 ± 2.06) (*p* < 0.001) in the case group, as shown in Figure 5a and Table 2. We did not find a statistically significant difference in the percentage of eosinophil cells between the case and control groups at T0; this difference was found at T1 (*p* < 0.001), as shown in Figure 5a and Table 2. The Cilateted/Goblet cells (C/G ratio) in the case group showed a statistical significance at T0 (1.31 ± 0.68) and T1 (2.72 ± 1.34) (*p* < 0.001), as shown in Figure 5b and Table 1. We did not find a statistically significant difference in the C/G ratio between the case and control groups at T0, while this difference was found at T1 (*p* < 0.001) (Figure 5b and Table 2).

**Figure 6:**
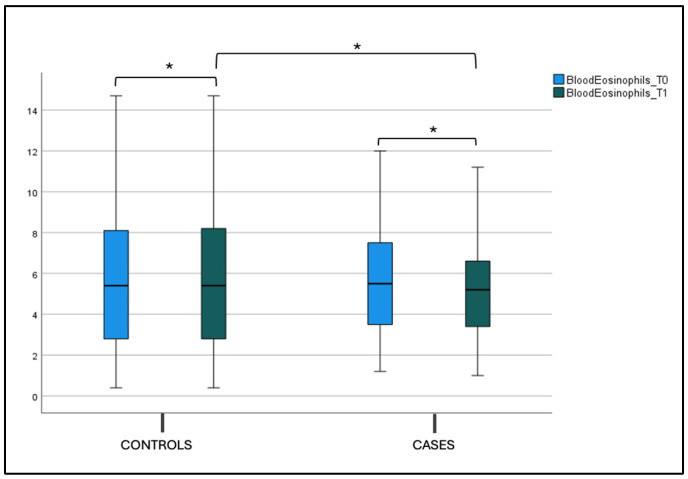
Blood eosinophils count at T0 and T1 in the case and control groups. Box plot representing eosinophils values at T0 and T1 in both the case and control groups (* not significant).

**Figure 7:**
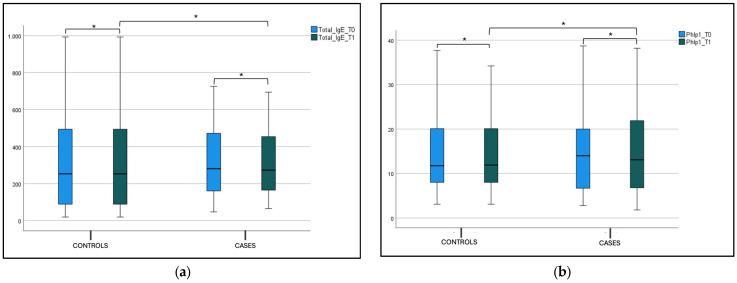
(**a**) Total IgE (tIgE) level at T0 and T1 in the case and control group. Box plot representing total IgE (tIgE) level at T0 and T1 in both case and control group (* not significant). (**b**) Phlp1 level at T0 and T1 in the case and control group. Box plot representing Phlp1 level at T0 and T1 in both case and control group (* not significant).

No statistical difference was found in the blood eosinophils count, total IgE (tIgE), and Phlp1 values in the control and case groups at T0 and T1 in both the intergroup and intragroup variability (Figure 6, Figure 7a,b and Table 2).

## 5. Discussion

The encouraging findings of this study demonstrate that the addition of Quertal^®^ to antihistamine therapy significantly improved both subjective and objective markers of mild/moderate AR in children. Specifically, the combination of Quertal^®^ with antihistamines resulted in significant reductions in both nasal and exhaled nitric oxide levels (nFeNO and eFeNO), which are widely recognized markers of allergic inflammation. Additionally, the treatment group also saw a marked decrease in nasal eosinophil counts, further validating Quertal’s role as an anti-inflammatory agent.

After three months of treatment, patients in the Quertal^®^ group exhibited a 30% reduction in nFeNO levels from baseline, compared to a 5% decrease in the control group. Likewise, eFeNO levels dropped by 25% in the Quertal^®^ group, significantly reducing allergic inflammation. Nasal eosinophil counts were also notably reduced by 40%, suggesting that the nutraceutical may be influential in managing eosinophil-mediated inflammation. Nasal eosinophil counts were also reduced by 40%, suggesting that the nutraceutical may influence eosinophil-mediated inflammation. The significant improvement in the C/G ratio confirms the amelioration of nasal cellularity [31,32]. Goblet cell hyperplasia is a hallmark of AR, characterized by an increased number of goblet cells in the nasal mucosa. This increase is associated with the inflammatory response and tissue remodeling processes, including eosinophil infiltration and epithelial damage [33]. In addition, post-therapy, the mean nasal flow improved by 20%.

The choice of cetirizine or levocetirizine as the antihistamine in this study plays a practical role in the observed outcomes. These second-generation antihistamines, in addition to being widely available, are well known for reducing symptoms such as nasal congestion, sneezing, and itching, exacerbated by grass pollen allergens [34,35,36,37]. The synergistic effect between antihistamines and Quertal^®^, mainly in reducing nasal eosinophil recruitment and cytokine release, likely accounts for the significant improvements in both nFeNO and eFeNO levels in the case group.

The specific formulation of Quertal^®^ may significantly contribute to this synergistic effect, particularly considering that the lipid matrix based on medium-chain triglycerides in the slow-release layer improves the bioavailability of Quercetin. However, the inherent challenges of Quercetin’s rapid metabolism, low solubility, and efflux mechanisms that limit its overall bioavailability still need a thorough examination. In the case of *Perilla frutescens*, it may contribute to its anti-inflammatory and anti-allergic effects, but proper standardization would ensure that each supplement dose contains an adequate and predictable amount of its compounds, and their bioavailability would be adequately addressed.

In this context, the decision to exclude intranasal corticosteroids played a crucial role in isolating the specific effects of Quertal^®^ because their potent anti-inflammatory effect could alter the results of nasal cytology. Additionally, by avoiding intranasal corticosteroids that significantly reduce nitric oxide (NO) levels, the study was able to demonstrate that the supplement itself may substantially reduce inflammatory markers like nFeNO and eFeNO.

**Figure 8:**
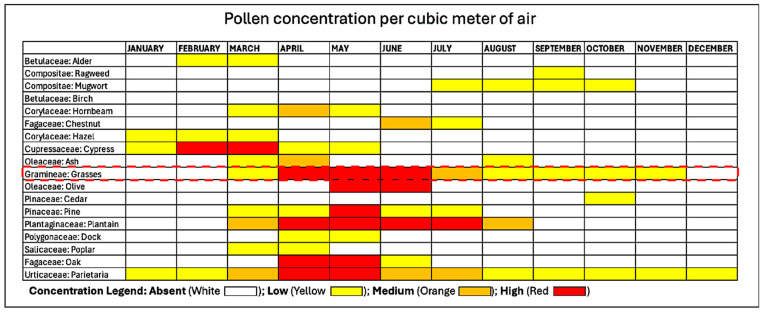
Pollen calendar of the metropolitan area of Rome. The red dotted line encloses Grass Pollen concentration, which our patients were exclusively sensitized to, in the metropolitan area of Rome from which all our patients resided. The pollen calendar was developed by processing a multi-year data series collected by the Aerobiological Monitoring Center of the University of Rome Tor Vergata. For each family, three concentration levels are indicated: low, medium, and high, represented by yellow, orange, and red, respectively. The calendar is available at https://polline.uniroma2.it/calendario-pollinico-della-citta/. Accessed on 15 December 2022.

Monosensitization to Phleum pratense (Timothy grass), one of the most prevalent allergens in urban areas like Rome, was considered to further target the nutraceutical’s effect. The well-defined pollination season of grass pollen, peaking in spring to early summer, allowed us to focus the study on a specific period when AR symptoms are at their onset until most pronounced, as demonstrated in Figure 8. In our opinion, this approach ensured a consistent timeframe for evaluating a preventive effect in reducing symptoms and treatment efficacy, while avoiding confounding factors such as new sensitization to overlapping pollination.

Our results align with previous studies by including pediatric patients and focusing on subjective (NSS) and objective (AAR, nasal cytology) markers, providing a more comprehensive view of Quertal^®^’s benefits. Unlike previous studies that primarily relied on subjective symptom relief, our work emphasizes quantifiable inflammatory markers, offering a more robust evaluation of the nutraceutical’s effects.

Many studies attest to the effectiveness of the association of these molecules in the management of the clinical symptoms of AR and in reducing the need for drugs in the pediatric age.

Through such an Italian clinical trial, it was demonstrated in the first phase that the multicomponent nutraceutical (Lertal^®^) improved the effect of antihistamine treatment with an average reduction of about 64% in AR symptoms’ severity, thus possibly reducing the onset of clinical relapses with savings on drug use. The second phase of the same trial, a parallel-group extension study lasting 4–12 weeks, in which cases continued treatment with the nutraceutical tablets, confirmed the favorable effects of the first phase, halving the risk of AR exacerbation after one month of antihistamine treatment and with a preventive effect towards clinical exacerbations [38,39]. There were also demonstrated changes over time in spirometry after a year, a result that could mean a potential prophylactic impact towards the possible onset of asthma in patients with AR. In our study, spirometric data (FEV1) at baseline and post-bronchodilation did not present statistically significant differences. However, the enrolled patients had no clinically evident asthma symptoms, and the spirometric values were average, so we did not find any changes in the pre- and post-therapy spirometric values. However, in general, better control of AR can significantly improve the spirometric picture, as well as the bronchial inflammation, as seen in our group of patients by the significant reduction in eFeNO values. So, a reasonable control of the AR improves the eFeNO values, even in patients who did not have positive values of the spirometric parameters and, therefore, who present a chronic basal bronchial inflammation not yet clinically manifest with positive spirometry [25,40].

Another study recently evaluated the use of the same nutraceutical component as adjunctive therapy in children affected by AR, showing it to help reduce antihistamine use in the case group in comparison with the control group under standard treatment only [41].

The study conducted by Ariano tested 23 adults with positive SPTs to Parietaria to evaluate the efficacy of the Lertal^®^ on symptoms of seasonal allergic rhinitis and the consumption of anti-allergic drugs. The improvement of nasal symptoms was tested using the Total Symptoms Score at the first (baseline) and second (final) visit. The conclusion showed an apparent efficacy of Lertal^®^ in reducing nasal symptoms (70% symptoms, 73% use of antiallergic drugs, *p* < 0.001). This activity was objectively confirmed by reducing the consumption of anti-allergic medications to relieve symptoms. No side effects were reported. The critical lack of such a study was the control group, and the authors do not explain in detail what type of anti-allergic therapies are carried out [42]. Similar results of a reasonable control of AR symptoms were found in our study.

A recent study evaluated the possible change in spirometry in two groups of children affected by AR: The first group under a nutraceutical (Lertal^®^), and the second one was the control group. The children were visited at baseline and the end of the nutraceutical treatment after one year. The results confirmed that children in the case group had a significantly higher MEF50 than the control group. In conclusion, the study demonstrated that a course with a multicomponent nutraceutical could prevent the decline of MEF50 in children with AR [43].

Instead, no changes were detected in blood parameters, such as blood eosinophil count, tIgE, and sIgE for Phlp1. A more prolonged study duration could have provided significant variations within the laboratory parameters.

Regarding blood parameters, a recent study conducted in a mouse model with AR using the ionic cross-linking method for intranasal administration of quercetin (QCS) demonstrated that QCS treatment significantly reduced the number of sneezes and nasal rubs in mice with AR while reducing the levels of inflammatory factors such as immunoglobulin E (IgE), interleukin (IL)-17, tumor necrosis factor (TNF)-α, and (IL)-6 to relieve the AR symptoms. Furthermore, thanks to hematoxylin-eosin (HE) staining, improvement of a damaged nasal mucosa has been demonstrated. These experimental results suggest that QCS can effectively suppress allergic inflammation in a mouse model and represent a promising therapeutic option for AR in humans [44]. Another recent study on a murine model was published on the anti-allergic biological activity of Quercetin in AR, where the authors aimed to explore the effects of Quercetin on the balance of T helper type 1 (Th1)/Th2 cells and regulatory T cells (Treg)/Th17. The results showed that Quercetin alleviates nasal itching and sneezing from a clinical point of view. It was also assessed through a laboratory evaluation that Quercetin reduced IgE and IgG1 and increased IgG2 in serum, inactivating the NF-κB pathway. Taken together, Quercetin attenuated AR symptoms by balancing the Th1/Th2 and Treg/Th17 ratios and inactivating the NF-κB pathway. Therefore, these results suggested that Quercetin can be used to treat AR [45]. Beyond nutraceuticals, several other adjuvant therapies are being explored to enhance the management of allergic rhinitis by modulating the immune response and reducing inflammation. Among these, probiotics have gained increasing attention for their potential immunomodulatory effects, with recent studies suggesting improvements in symptom control and quality of life in patients with allergic rhinitis. A systematic review and meta-analysis concluded that probiotics might improve symptoms and quality of life in AR patients, though the evidence is limited due to study heterogeneity [46]. Another review highlighted the positive effects of probiotic supplementation in treating AR, noting improvements in quality of life and modulation of the inflammatory response [47]. Furthermore, a meta-analysis focusing on pediatric patients demonstrated that probiotics effectively and safely improved AR symptoms and quality of life, though they did not prevent the onset of AR [48]. Recent findings also suggest that probiotics may reduce inflammatory markers and improve symptom control by shifting the immune balance towards a more tolerogenic profile [49]. All these findings highlight the growing interest in complementary approaches, such as probiotics and nutraceuticals, and several adjuvant therapies in optimizing allergic rhinitis management. While probiotics have demonstrated potential immunomodulatory effects, our study provides further objective evidence supporting the role of Quertal^®^ as an effective add-on therapy for pediatric allergic rhinitis, showing significant benefits in reducing nasal inflammation and improving symptom control.

### 5.1. Strength of This Study

The strengths of this work are several. First, it is a unique study conducted in pediatric age that analyzes children affected by seasonal AR, monosensitised to grass pollens from a laboratory (eosinophils, tIgE, and sIgE for Phlp1), functional (nasal and exhaled nitric oxide, nasal cytology), subjective (NSS), and objective (AAR, spirometry) perspective. Furthermore, this work, in light of the encouraging results obtained, underlines the valuable role of specifically formulated nutraceuticals as an add-on therapy for improving AR symptoms with a high safety profile. Lastly, the sample size is sufficiently large to make possible a valid statistical analysis. The findings of this study are particularly relevant to pediatric patients with mild-to-moderate allergic rhinitis who are monosensitized to grass pollen. However, caution should be taken when generalizing these results to populations with multiple sensitizations or severe disease requiring corticosteroids. Future studies should explore whether these benefits extend to broader populations, including adults or patients with more severe allergic profiles.

### 5.2. Limitations

While this study provides valuable insights, several limitations should be acknowledged. First, the study duration of three months may not have been sufficient to detect long-term changes in laboratory parameters such as total IgE and specific IgE levels. A longer follow-up would allow for a better assessment of whether Quertal^®^ has sustained effects on systemic inflammation markers. Additionally, this study relied on patient-reported symptom scores, which, while commonly used in clinical practice, introduce an element of subjectivity. This could be addressed in future studies by incorporating more objective measurements, such as daily nasal airflow monitoring.

Furthermore, patient selection may have introduced potential biases. The exclusion of patients with severe AR or those who required intranasal corticosteroids means the results are more applicable to patients with mild to moderate AR and may not be generalizable to those with more severe symptoms. The absence of a placebo group also limits the ability to isolate Quertal^®^’s effect completely. However, using antihistamines as a control offers a reasonable comparison for current clinical practice. In conclusion, the specific formulation of Quertal^®^, containing a dry extract of Perilla rather than active components such as Quercetin and vitamin D3, does not allow for a clear understanding of its efficacy, affecting the generalizability of the results.

### 5.3. Future Research Directions

To build on the findings of this study, future research should focus on larger cohorts and more extended treatment periods to fully assess the long-term effects of Quertal^®^. Expanding this study to include different populations, such as adults or patients with more severe AR, would provide more comprehensive data on its effectiveness across a broader range of patients. Additionally, conducting a placebo-controlled trial would provide more robust evidence of the true benefit of Quertal^®^ in managing AR.

Future studies could also investigate the potential role of Quertal^®^ as part of a maintenance therapy strategy to prevent exacerbations during peak pollen seasons. Assessing its use in conjunction with allergen-specific immunotherapy (AIT) or its impact on delaying the onset of asthma in pediatric patients with AR could offer new preventive strategies in allergy management.

## 6. Conclusions

This study highlighted that the nutraceutical Quertal^®^, used in addition to antihistamine therapy in a cohort of pediatric patients affected by AR, appears to induce significant benefits in nasal obstruction, reducing symptoms and alleviating local allergic inflammation from a subjective, objective, and functional point of view.

From a clinical perspective, this combined approach may benefit children with moderate symptoms who are not fully managed by antihistamines alone. One important implication for clinical practice is the potential to reduce the need for higher doses of antihistamines, thereby minimizing side effects commonly associated with such therapies, such as drowsiness and cognitive impairment.

Additionally, Quertal^®^’s favorable safety profile makes it a compelling option for long-term use in pediatric patients. While the three-month treatment period provided encouraging results, extending therapy through peak pollen seasons or over a year could offer more significant insights into its prophylactic potential. Future clinical guidelines might consider incorporating nutraceuticals for long-term management, especially in patients with mild to moderate AR.

Given the chronic nature of AR and the potential for symptom persistence into adulthood, using a nutraceutical that offers both anti-inflammatory and immunomodulatory effects may help reduce the progression of allergic symptoms and possibly delay or prevent the onset of comorbid conditions like asthma.

## Figures and Tables

**Table 1:** Characteristics of the study population. Allergic Rhinitis (AR).

Characteristics	Mean ± SD	*p*-Value
Age (years)		
- Control	9.52 ± 2.81	*p* = 0.653
- Case	8.99 ± 3.08	
Weight (kg)		
- Control	35.67 ± 13.94	*p* = 0.573
- Case	34.15 ± 13.01	
Height (cm)		
- Control	135.76 ± 16.75	*p* = 0.423
- Case	133.06 ± 16.80	
BMI (kg/m^2^)		
- Control	18.59 ± 3.33	*p* = 0.938
- Case	18.64 ± 3.63	
Characteristics	Count	Percent
Sex (Female)		
- Control	18	36%
- Case	20	40%
AR		
- Control	50	100%
- Case	50	100%
Asthma		
- Control	22	44%
- Case	21	42%

**Table 2:** Comparison of intragroup and intergroup values related to the considered variables between the case and control. Mean Nasal Flow (mNF); Nasal Nitric Oxide (nFeNO); Forced Expiratory Volume in the First Second (FEV1); Exhaled nitric oxide (eFeNO); and ratio of ciliated cells to goblet cells (C/G Ratio).

Characteristic	Value				*p*-Value Intragroup
	T0		T1		
	Mean ± SD	*p*-Value Intergroup	Mean ± SD	*p*-Value Intergroup	
Nasal Symptom Score (NSS)					
- Control	22.10 ± 4.76	*p* = 0.911	19.90 ± 5.02	*p* = 0.001	*p* = 0.001
- Case	22.00 ± 4.12		13.38 ± 3.61		*p* < 0.001
mNF (%) pre-hydrazine					
- Control	67.42 ± 25.98	*p* = 0.957	71.31 ± 24.92	*p* = 0.041	*p* = 0.045
- Case	67.14 ± 26.18		80.60 ± 20.34		*p* = 0.001
mNF (%) post-hydrazine					
- Control	74.25 ± 22.53	*p* = 0.872	77.13 ± 23.25	*p* = 0.018	*p* = 0.031
- Case	74.95 ± 20.63		86.72 ± 15.81		*p* = 0.001
Nasal FeNO (nFeN0)					
- Control	1275 ± 660.69	*p* = 0.985	1280 ± 641.10	*p* = 0.001	*p* = 0.802
- Case	1277.5 ± 697.21		798.10 ± 436.21		*p* = 0.001
preFEV1					
- Control	99.70 ± 9.38	*p* = 0.763	99.92 ± 9.53	*p* = 0.601	*p* = 0.322
- Case	100.30 ± 10.46		101.02 ± 11.37		*p* = 0.104
Post FEV1					
- Control	103.24 ± 9.27	*p* = 0.920	103.78 ± 8.40	*p* = 0.423	*p* = 0.382
- Case	103.44 ± 10.54		105.48 ± 12.34		*p* = 0.067
Exhaled FeNO					
- Control	14.88 ± 5.43	*p* = 0.987	14.89 ± 4.46	*p* < 0.001	*p* = 0.979
- Case	14.86 ± 3.79		10.33 ± 3.36		*p* < 0.001
Blood Eosinophils					
- Control	5.65 ± 3.61	*p* = 0.993	5.64 ± 3.60	*p* = 0.625	*p* = 0.771
- Case	5.64 ± 2.73		5.34 ± 2.50		*p* = 0.121
Nasal Eosinophils					
- Control	9.00 ± 3.64	*p* = 0.739	9.10 ± 3.96	*p* = 0.001	*p* = 0.795
- Case	9.20 ± 2.16		6.20 ± 2.06		*p* = 0.001
Total IgE, Ku/L					
- Control	322.27 ± 271.81	*p* = 0.992	322.18 ± 271.77	*p* = 0.771	*p* = 0.122
- Case	322.73 ± 192.74		308.88 ± 172.79		*p* = 0.058
C/G Ratio					
- Control	1.30 ± 0.60	*p* = 0.938	1.29 ± 0.55	*p* = 0.001	*p* = 0.678
- Case	1.31 ± 0.68		2.72 ± 1.34		*p* = 0.001
Phl p1					
- Control	14.77 ± 9.75	*p* = 0.583	14.63 ± 9.15	*p* = 0.968	*p* = 0.486
- Case	15.89 ±10.53		14.70 ± 9.03		*p* = 0.093

## Data Availability

The original contributions presented in this study are included in the article/Supplementary Material. Further inquiries can be directed to the corresponding author(s).

## Supplementary Materials

The following supporting information can be downloaded at: https://www.mdpi.com/article/10.3390/jcm14051517/s1, Table S1: Consolidated Standards of Reporting Trials (CONSORT) completed checklist [50].
