# Supplementary material for: Synergic Efficacy of a Multicomponent Nutraceutical Add-On Therapy in Seasonal Allergic Rhinitis in Children: A Prospective, Randomized, Parallel-Group Study
Source: J Clin Med. 2025 Feb 24;14(5):1517. doi: 10.3390/jcm14051517 (PMC11900512; doi:10.3390/jcm14051517)
Supplement: Supplementary file 1 [file jcm-14-01517-s001.zip › jcm-3468468-supplementary.pdf]

Table S1. CONSORT 2010 checklist of information to include when reporting a randomised trial*

Section/Topic	Item No	Checklist item	Reported on page No
**Title and abstract**			
	1a	Identification as a randomised trial in the title	LINE 2-4
	1b	Structured summary of trial design, methods, results, and conclusions (for specific guidance see CONSORT for abstracts)	LINE 9-24
**Introduction**			
Background and objectives	2a	Scientific background and explanation of rationale	LINE 26-80
	2b	Specific objectives or hypotheses	LINE 81-86
**Methods**			
Trial design	3a	Description of trial design (such as parallel, factorial) including allocation ratio	LINE 89, 115-128
	3b	Important changes to methods after trial commencement (such as eligibility criteria), with reasons	NO
Participants	4a	Eligibility criteria for participants	LINE 101-105
	4b	Settings and locations where the data were collected	LINE 92-94
Interventions	5	The interventions for each group with sufficient details to allow replication, including how and when they were actually administered	LINE 129-144
Outcomes	6a	Completely defined pre-specified primary and secondary outcome measures, including how and when they were assessed	LINE148-156
	6b	Any changes to trial outcomes after the trial commenced, with reasons	NO
Sample size	7a	How sample size was determined	LINE 243-247
	7b	When applicable, explanation of any interim analyses and stopping guidelines	NOT APPLICABLE
Randomisation:			
Sequence generation	8a	Method used to generate the random allocation sequence	LINE 115-128
	8b	Type of randomisation; details of any restriction (such as blocking and block size)	LINE 120-122
Allocation concealment mechanism	9	Mechanism used to implement the random allocation sequence (such as sequentially numbered containers), describing any steps taken to conceal the sequence until interventions were assigned	LINE 127-130
Implementation	10	Who generated the random allocation sequence, who enrolled participants, and who assigned participants to	LINE 125

		interventions	
Blinding	11a	If done, who was blinded after assignment to interventions (for example, participants, care providers, those assessing outcomes) and how	NOT APPLICABLE (not blinded)
	11b	If relevant, description of the similarity of interventions	NOT APPLICABLE (open-label study)
Statistical methods	12a	Statistical methods used to compare groups for primary and secondary outcomes	LINE 247-255
	12b	Methods for additional analyses, such as subgroup analyses and adjusted analyses	Not performed
**Results**			
Participant flow (a diagram is strongly recommended)	13a	For each group, the numbers of participants who were randomly assigned, received intended treatment, and were analysed for the primary outcome	TABLE 1
	13b	For each group, losses and exclusions after randomisation, together with reasons	LINE 115-116
Recruitment	14a	Dates defining the periods of recruitment and follow-up	LINE 94
	14b	Why the trial ended or was stopped	The trial was completed as planned; no early termination occurred.
Baseline data	15	A table showing baseline demographic and clinical characteristics for each group	TABLE 1
Numbers analysed	16	For each group, number of participants (denominator) included in each analysis and whether the analysis was by original assigned groups	TABLE 1
Outcomes and estimation	17a	For each primary and secondary outcome, results for each group, and the estimated effect size and its precision (such as 95% confidence interval)	RESULT SECTION AND FIG2-5
	17b	For binary outcomes, presentation of both absolute and relative effect sizes is recommended	Not applicable (continuous variables were used).
Ancillary analyses	18	Results of any other analyses performed, including subgroup analyses and adjusted analyses, distinguishing pre-specified from exploratory	NOT APPLICABLE
Harms	19	All important harms or unintended effects in each group (for specific guidance see CONSORT for harms)	LINE 274-275
**Discussion**			
Limitations	20	Trial limitations, addressing sources of potential bias, imprecision, and, if relevant, multiplicity of analyses	LINE 457-474

Generalisability	21	Generalisability (external validity, applicability) of the trial findings	LINE 456-461
Interpretation	22	Interpretation consistent with results, balancing benefits and harms, and considering other relevant evidence	LINE 378-447
**Other information**			
Registration	23	Registration number and name of trial registry	NOT APPLICABLE
Protocol	24	Where the full trial protocol can be accessed, if available	Affiliation n.1
Funding	25	Sources of funding and other support (such as supply of drugs), role of funders	NO, LINE 519

Citation: Schulz KF, Altman DG, Moher D, for the CONSORT Group. CONSORT 2010 Statement: updated guidelines for reporting parallel group randomised trials. BMC Medicine. 2010;8:18.
© 2010 Schulz et al. This is an Open Access article distributed under the terms of the Creative Commons Attribution License (http://creativecommons.org/licenses/by/2.0), which permits unrestricted use, distribution, and reproduction in any medium, provided the original work is properly cited.

*We strongly recommend reading this statement in conjunction with the CONSORT 2010 Explanation and Elaboration for important clarifications on all the items. If relevant, we also recommend reading CONSORT extensions for cluster randomised trials, non-inferiority and equivalence trials, non-pharmacological treatments, herbal interventions, and pragmatic trials. Additional extensions are forthcoming: for those and for up-to-date references relevant to this checklist, see www.consort-statement.org.
